# Anatomical Consideration of Brain Syphilis, with Report of Three Cases1Read at the twenty-ninth annual meeting of the Medical Association of Central New York, at Rochester, October 20, 1896.

**Published:** 1897-04

**Authors:** William C. Krauss

**Affiliations:** Professor of nervous diseases, Medical Department of Niagara University, Buffalo, N. Y.; Fellow of the American Neurological Association, 371 Delaware Avenue


BUFFALO MEDICAL JOURNAL.

Vol. XXXVI. APRIL, 1897. No. 9.

### Original Communications.

### ANATOMICAL CONSIDERATION OF BRAIN SYPHILIS, WITH REPORT OF THREE CASES.[1]

By WILLIAM C. KRAUSS, M. D.,
Professor of nervous diseases, Medical Department of Niagara University, Buffalo, N. Y.;
Fellow of the American Neurological Association.

WHILE the clinical picture of syphilis of the brain may be a varied and highly-colored one, the anatomical easel upon which it rests is quite simple and uncomplicated.

It is my purpose today to consider the anatomical bearings of brain syphilis and to illustrate the same by several clinical cases which have recently come under my observation. As a rule, syphilis attacks the brain in one of three different ways: First, by setting up an inflammatory process in the walls of the arteries, more especially those situated at the base of the brain; second, by the formation of gummatous tumors in the cortical layer or at the base of the brain; third, by developing a meningitis through exudation and inflammation, continuing until the neighboring cortical surfaces become involved, assuming a form of meningo-encephalitis.

I shall omit all reference to the degenerative processes affecting the brain exampled by dementia-paralytica—where the dictum of syphilitic infection is still debatable, and hence uncertain—also those cases of multiple cerebro-spinal syphilis.

The process involved in these three above-mentioned lesions is identical, the underlying cause being, no doubt, a bacillus of which no positive knowledge is as yet obtainable. The bacillus described by Lustgarten, in 1884, has been found in syphilitic sores and condylomata, but no satisfactory experimental evidence has yet been obtained, and until pure cultures and other proofs are brought forward not much reliance can be placed on this organism. The bacillus of Eve and Lingard, described in 1886; that of Disse and

1. Read at the twenty-ninth annual meeting of the Medical Association of Central New York, at Rochester, October 20, 1896.

Taguchi, also discovered in 1886, and that of Golasz, described in 1894, are all open to the same criticism as Lustgarten's, and the true bacillus must remain a suppositious factor until its identity shall be satisfactorily proven.

Subsequent to the activity of the bacillus irritation, there follow inflammation, cell-proliferation, new growth of vascular tissue, tissue growth, and then a degenerative process and disintegration.

Heubner, in 1874, first described accurately the changes taking place in the arteries, especially at the base of the brain, resulting in an arteritis or endarteritis, and found existing either as an independent disorder or as part of a local syphilitic affection. An endarteritis is found, due to the pouring out of round cells from the *vasa vasorum*, an endothelial proliferation, followed by thickening of the intima, fenestrated membrane and adventitia. As a result, the lumen of the vessel is narrowed, even occluded, or the intima becomes roughened and changed and a thrombus forms, which may in turn give rise to an embolus, or through the weakening of the muscular coat of the arteries minute aneurisms may form and their rupture cause hemorrhages. Two serious brain lesions may follow such an arteritis—first, softening due to the thrombus or embolus, and secondly, destruction and pressure upon the brain tissue by the hemorrhage.

An interesting medico-legal case of the latter came under my observation about three years ago, briefly narrated as follows:

F. S., aged 35 years, Swedish extraction; sailor by occupation; had sailed the lakes for seven or eight years, making his home at Buffalo. He was in the habit of spending his nights when on shore at a notorious dance hall in the infected district. One night he chanced to meet a woman to his liking, who was a singer in the resort, and whose husband was "the strong man," doing certain tricks, as stone-breaking, tearing chains asunder and the like. The couple proceeded up-stairs to a private room, drank heavily of strong liquors, and, if the report of the woman can be believed, the sailor made unsuccessful attempts at her person. Leaving the room and descending the stairs they met the husband, and on being informed of what had transpired he struck the sailor on the jaw, felling him, and in a few moments expired. Post-mortem examination of the cranial cavity revealed a large flattened clot of blood in the posterior fossa of the cranium—the same having escaped from a large opening in the basilar artery, near its bifurcation into the posterior cerebrals. The basilar artery was the seat of an arteritis, also endarteritis, with thickening of the lumen in some places and thinning in others. It was at one of the thinned portions where the rupture occurred. Other evidences of cerebral or arterial disease were wanting, and the anatomical diagnosis was reported as "cerebral basilar hemorrhage."

Of course, no history of syphilis could be obtained, but the seat of disease, condition of the artery, occupation, habits and surroundings of the man, leave little doubt as to some previous syphilitic inoculation. No doubt the woman's husband was, to a great degree, a victim of circumstances. With the degenerated condition of the arteries, as was found in the case, the sexual excitement might of itself have produced the arterial rupture, and this, further accentuated by the increased arterial tension due to the large amount of stimulants which he had taken, would have required but a very slight shock, either mental or physical, to have produced a disruption of the diseased vessel. The force of the blow was of itself not so important under these circumstances, although at the trial much stress was laid on the assumption that the prisoner must have dealt a terrible knock-out blow, being noted for his strength, which, in fact, consisted only in stage tricks, being actually possessed of only ordinary strength.

Syphilitic inflammation of the brain resulting in gummatous tumors or syphilomata is of quite common occurrence, and is the most characteristic lesion of syphilis of the nervous system. Such tumors are generally situated at the base of the brain, less frequently upon the convexity of the frontal and parietal convolutions. They are scarcely ever deep-seated, but are found taking their origin generally from the pia, but may also originate from the arachnoid, dura or blood-vessels. They are irregular in shape, varying in size from a pea to a walnut. As usually met with, they are moderately firm yellowish-white nodules, having on section an appearance suggestive of the cut surface of a horse-chestnut.

Anatomically, the periphery consists of translucent fibrous-looking tissue, intimately associated with the neighboring structures, so that enucleation is impossible, or a dense infiltration of small spheroidal cells, while the center is in a state of cheesy degeneration. These growths are sometimes associated with a localised meningitis, also a specific inflammation of the arteries.

An interesting case of syphiloma came under my notice two years ago, which, although not verified by autopsy, was nevertheless so evident as to leave no room for doubt:

A. B., female ; age, 23 ; weight, 85 pounds ; height, 5 feet 1 inch ; married when 19 years old ; has no children, but gives history of two miscarriages. Her husband, a worthless scamp, acquired syphilis some three years before his marriage, but never underwent any systematic treatment. The patient became infected soon after marriage, showed secondary manifestations within a year, and when 22 years old began to experience symptoms pointing to cerebral implication. She complained bitterly of severe occipital headaches, boring and bursting in character, more intense at night, with slight intermissions during the day. Nausea and vomiting, with dizziness and loss of vision, soon appeared, the latter increasing until she became totally blind. There were also present other symptoms denoting some basal disturbance, as double vision previous to the total blindness, ptosis and paresis of all the extremities.

I was called in consultation by Dr. Seeley, of Attica, N. Y., to see her about six months after the beginning of the cerebral symptoms and found her condition as follows : A small, emaciated young woman, in bed, suffering with intense occipital headaches, dizziness, nausea and vomiting at irregular intervals during the day and night. Head is sensitive to percussion, more especially over the occiput. Eyes : orbits are convergent, especially the right ; slight bilateral ptosis ; pupils dilated and eyes have a glassy appearance. Ophthalmoscopic examination shows optic atrophy; vision of both eyes is *nil ;* tongue shows no deviation, but is thickly coated ; olfaction and audition are unimpaired ; sensation of the face undisturbed. Arms : muscles of the arms are much wasted; no contractures; tendon reflexes exaggerated; muscular strength almost gone. Legs : limbs are also much atrophied ; patellar reflexes greatly exaggerated ; ankle clonus is present ; strength of the legs is almost entirely lost ; is unable to stand on her feet, but can extend the leg slightly on the thigh. Urine is increased in quantity, but contains neither albumin nor sugar ; the bladder and rectal sphincters are weakened.

After hearing the history of the case, there remained no doubt as to the diagnosis of cerebral syphilis, being in all probability a gummatous tumor, situated in the cephalic portion of the pons, or in the intercrural space, and extending into both crura cerebri.

The location of the gumma was based upon the presence of a diplegia, with exaggerated reflexes, and symptoms denoting a lesion along the course of the oculo-motorius nerves.

The patient died two weeks after my examination, and autopsy was refused.

The third variety of brain syphilis—gummatous meningitis or meningo-encephalitis—affects both the cortical and basal membranes, the latter, however, much more frequently. The gummatous tissue forms a diffused gray gelatinous layer covering a considerable extent of the surface or base of the brain. The membranes over the convexity are thickened, matted together by gummatous tissue, and the brain surface beneath is softened.

The pathology of the process is well described by Ziegler as follows:

The first thing observed in the meninges is a small patch of inflammation which soon leads to the formation of a gray, or grayish-red, semi-translucent or gelatinous knot of granulation tissue. In the earlier stages the tissue of the knot is extremely cellular and contains a number of new-formed capillaries. As the process goes on, some of the granulation tissue becomes fibro-cellular and some undergo caseation. The adjacent brain tissue seldom or never remains intact, the inflammation extending into the cortex along the pial sheaths of the vessels, also directly. When arterial branches pass through these granulomatous foci they are speedily infected, resulting in endarteritis. The same fate awaits the nerve fibers passing through the inflamed region and undergo inflammatory infiltration, and afterwards becoming enclosed and beset by coarse fibrous tissue they speedily atrophy and break down. The smaller foci can undoubtedly disappear by reäbsorption, and the thickened meninges may present the appearance of an old simple inflammation.

This form of brain syphilis is most amenable to treatment, as the following case will show, but such treatment must be heroical, methodical and persistent:

Annie C., age, 25 years; height, 5 feet 4 inches; weight, 120 pounds; complexion, dark; constitution, rather frail. Father and mother are living and healthy; grandparents lived to old age. There is no history of syphilis, tuberculosis or cancer obtainable. Never had any of the diseases of infancy and was always in the best of health. In 1891 she committed an indiscretion, ran away from home and lived for four years in a sporting house. In September, 1891, she acquired syphilis and was treated for primary and secondary symptoms.

In September, 1895, she first began to have pains in the head, sharp, shooting and continuous, beginning at the vertex and radiating downward in all directions. The head was tender and sensitive and felt as if "something were growing inside of it." She complained much of nausea and vomited occasionally. Bowels were loose; sleep much disturbed. Her physician prescribed mercurial preparations and potassium iodide. The headaches not diminishing, I was called in consultation by Dr. Briggs, of Buffalo, November 10, 1895, and found a thin, pale, emaciated young woman, whose body and extremities were covered with the characteristic roseola of syphilis, suffering with excruciating pains "all over the head." She and her husband claim that the eruption followed the administration of mercury. The head was very sensitive on pressure, and was very tender and sore over the nucha. Temperature was normal; pulse was 80. I advised mercurial inunctions and the protoiodide of mercury pills, ½ grain, three times daily. After the fourth inunction, salivation appeared, and immediately the head pains began to diminish, so that in three days the head was entirely free from pain and she was able to be about attending to her duties. The inunctions were discontinued, but the protoiodide pills were taken regularly. She was practically in good health for six weeks ; when as the rash began to disappear the headaches reappeared with greater intensity than ever before. They were accompanied by dizziness, tendency to fall to the right, nausea, vomiting and photophobia. She could not see to read or write and complained of pain in the eyeballs. On January 3, 1896, I was again called to see her and found her more emaciated and debilitated than at my first visit. As on the previous visit, she complained of the head pains, nausea and vomiting, unable to keep even water on her stomach, while vision was markedly diminished, and the room was heavily curtained on account of the photophobia. The acme of pain she referred to the left side of the head, over the frontal and parietal regions, with sensitiveness to percussion over this area. She has of late been misapplying words, calling for a spoon when she wanted a toothbrush, "cigarette" for a "glass of water," would call the nurse by the wrong name, and at times would not be able to make herself understood at all. The lower part of the face is slightly paretic. The eyes : ocular muscles not affected, as the orbits are freely movable in all directions ; the pupils are widely dilated, with some difference between the two sides. The fundus shows an acute papillitis (choked disc) on both sides. Audition, olfaction, gustation and sensibility of the face are unimpaired. Tongue protrudes, slightly deviated to the right. Extremities : there exists a paresis of the whole right side of the body, face, tongue, arm and leg. Dynamometer test : March 2d, right 24, left 29 ; March 6th, right 27.2, left 32 ; March 8th, right 30, left 34 ; March 15th, right 26, left 30 ; March 31st, right 31, left 35 ; April 10th, right 32, left 42. No disturbance of the general sensibility. Tendon reflexes are present, not exaggerated and without any perceptible difference between the two sides.

On March 3d, while on a couch, she complained of the right hand becoming numb and found she had no power over it ; hand was cold and finger nails were blue. On attempting to eat some bread and milk shortly afterwards, found she could not swallow ; it would choke and strangle her. On attempting to place her in bed, the right leg was also paretic and for some minutes she lost all power of speech, but was conscious and heard what the nurse was telling her. The whole attack lasted no longer than five minutes. Says the hand and leg felt drawn down forcibly.

March 4th she had a similar attack of paresis, affecting the right arm and leg, lasting only a minute. During the day and night the right arm and leg would contract forcibly, the arm flexed at elbow and wrist joints and adducted at shoulder ; the knee joint would be flexed and leg flexed on thigh. Her husband states that these spells occurred very often during the night and on two occasions awakened her.

March 8th—She is thoroughly salivated so that she can hardly speak. Saliva flows from her mouth. Is fed by enemata, and has been for four weeks.

March 25th—The pains are very much better ; there is no longer nausea ; appetite is good ; bowels are regular. Examination of the fundus shows the papillitis receding. The salivation still continues.

March 31st—The headaches are growing less in severity and are confined to the right side of the head. Vision is improving and the photophobia has disappeared. Has not vomited since March 21st ; appetite is good and bowels are regular.

She has been receiving one-half grain of morphia and one-sixth grain bichloride of mercury, hypodermatically, daily, since February 1st, along with tonics, enemata and a mouth wash.

She continued to improve daily, and on April 30th I ceased to see her daily, having asked her to come to my office once weekly for three months. The protoiodides were continued during this time, but not with much regularity. She was in the best of health, rode a bicycle and thoroughly enjoyed herself.

On September 1, 1896, not having seen her for three weeks, was called to see her and found the old trouble lighted up again. Headaches, nausea, vomiting, mental dulness, prostration and papillitis were again present. I determined to salivate her as speedily as possible, and injected fifteen minims of a solution of bichloride of mercury, one-third grain to ten minims, night and morning, along with inunctions of the yellow oxide ointment to the elbow and knee joints.

On September 10th salivation appeared and improvement began, which has continued until she is in fairly good health. As soon as the cerebral symptoms disappeared and her usual good health was expected to return, she began to complain of weakness of the hands and arms, tiredness in walking, and of pains in the elbow and knee joints. These persisted in spite of all manner of treatment. Examining the joints there was found no tenderness in the joints themselves, no pain on rubbing the joint surfaces together, no swelling, redness or increased temperature.

Assuming, therefore, that the pain was not articular or rheumatic, it occurred to me that a neuritic process might be present, due to the mercury, since the joints implicated were the ones which received the inunctions.

This proved to be the case, inasmuch as there existed great tenderness over the nerve points in the upper and lower extremities, marked weakness of the hands and a slight degree of leg drop. She complained of giving way of the hands, would let dishes fall to the floor, had difficulty in buttoning her clothes, and would stumble and trip while walking. The tendon reflexes were markedly diminished, the patellar reflexes hardly responding to the stimulus. Sensation was little if any affected. Trophic disturbances were also wanting. Electrical examination of the arm muscles showed quantitative changes only, the peroneal muscles acting in a similar manner.

The treatment directed towards the neuritis consisted of withdrawal of the mercurials, rest, galvanism, the administration of tonics and the iodides. Under this treatment the painful spots began to disappear and the muscle power slowly returned.

Analysis of this case shows it to be a specific meningo-encephalitis, affecting the frontal and parietal lobes of the left side, followed by a toxic neuritis. That it was not a gummatous tumor may be explained by the large extent of territory which it covered, reaching from the longitudinal fissure to the inferior frontal convolution, and perhaps extending to the base of the brain partly. The paresis of the right leg, arm and face, with aphasia and paraphasia; the history of several attacks of Jacksonian epilepsy, limited to the right side; the absence of disturbances of the cranial nerves, all point to the left cortical region as the seat of the disease. That the pathological process had not interfered permanently with the functions of the cortex is proven by the absence of all symptoms denoting brain destruction and the return of those which were but temporarily affected; as such may be mentioned the aphasia, the hemiparesis and the optic neuritis. These symptoms as presented plainly showed some lesion to the brain cortex, affecting the superficial layers only, either through pressure or, as is most probable, by a superficial inflammatory process of a specific nature.

The amount of mercury administered is almost incredible. Hypodermatic injections into the buttocks of about 1½ grains of the bichloride daily would be a toxic quantity even to the majority of syphilitics, but this amount injected daily for ten days shows to what extent mercury is tolerated by some of these cases. Along with these injections, during the first attack the hair was shaven closely over the left parietal region and inunctions of the yellow oxide ointment were made daily. During the second attack, these inunctions were made to the elbow and knee joints, inner surfaces, and here the toxic neuritis started.

Regarding the case as a desperate one on September 1, 1896, it was either heroic action or loss of the patient, and informing the husband of the results of mercurialisation, started with the injections as before mentioned. The iodides were not tolerated, even in very small doses, and their cessation was quickly determined upon. The ptyalism was not as severe and protracted during the second attack as the first and quickly subsided on withdrawal of the mercury and a mouth wash consisting mainly of tincture of myrrh.

*Prognosis.*—Cases of arteritis or endarteritis, with consequent embolus or thrombus formation, or disruption of the vessels, with destruction or compression of the brain tissues, are least liable to satisfactory treatment. While gummatous tumors, if small and located in the meninges, offer better advantages, larger syphilomata extending into the cortex, or affecting the base of the brain, cause destruction of neighboring brain tissue, and are to be reckoned in the same class, as regards results, with the sequelæ of endarteritis. In other words, complete, or even partial, recovery is extremely doubtful in all cases where the brain substance has been impinged upon, and the prognosis must be rendered with great caution. On the other hand, cases of meningitis, or meningo-encephalitis, offer much better results and a hopeful prognosis, dependent, of course, upon the thoroughness and audacity of the treatment.

371 Delaware Avenue.

